# No- and Low-Alcohol Wines: Perception and Acceptance in a Traditional Wine Region in Northern Italy

**DOI:** 10.3390/foods15010042

**Published:** 2025-12-23

**Authors:** Wasim Akhtar, Gavin Duley, Massimiliano Calvia, Edoardo Longo, Unais Sait, Emanuele Boselli

**Affiliations:** 1 Oenolab, NOI Techpark, Faculty of Agricultural, Environmental and Food Sciences, Free University of Bozen-Bolzano, Via A. Volta, 13/A, 39100 Bolzano, Italy; wasim.akhtar@student.unibz.it (W.A.); gavin.duley@unibz.it (G.D.); edoardo.longo@unibz.it (E.L.); 2 Faculty of Agricultural, Environmental and Food Sciences, Free University of Bozen-Bolzano, Piazza Università 5, 39100 Bolzano, Italy; massimiliano.calvia@unibz.it; 3 Faculty of Engineering, Free University of Bozen-Bolzano, Via Bruno Buozzi, 1, 39100 Bolzano, Italy; unais.sait@unibz.it; 4 International Competence Center on Food Fermentations, Faculty of Agricultural, Environmental and Food Sciences, Free University of Bozen-Bolzano, NOI Techpark, Via A. Volta, 13/A, 39100 Bolzano, Italy

**Keywords:** dealcoholized wine, low-alcohol wine, NoLo wines, consumer perception, social acceptance, purchase intention, Italy, consumer survey

## Abstract

The growing interest in no- and low-alcohol (NoLo) wines reflects evolving consumer preferences toward moderation, health, and mindful drinking. This study investigates consumer perception and acceptance of NoLo wines within a traditional wine context (Trentino-Alto Adige, Italy), based on a survey of 344 people. Respondents were primarily between 18 and 34 years old. Descriptive results indicated low familiarity and purchase frequency but positive attitudes, especially among women and health-oriented consumers. Nonparametric tests revealed that gender significantly affected familiarity, social acceptance, and willingness to recommend NoLo wines, with women respondents showing higher engagement and acceptance. Age showed a weaker but still significant effect on familiarity, while consumers who regularly consumed NoLo beverages exhibited greater social acceptance and willingness to recommend. In addition, logit and probit models suggested that preference for mid-to-low alcohol levels and prior experience with alcohol-free drinks positively influenced purchase frequency. In contrast, traditional wine consumption habits and expenditure had no significant effects. These findings suggest that while NoLo wine adoption in a premium wine region such as Trentino-Alto Adige is in an emerging phase, it is underpinned by evolving young consumer motivations toward moderation, well-being, and social inclusivity, offering clear opportunities for targeted market development and product innovation.

## 1. Introduction

There has been increasing consumer interest in no- and low-alcohol (NoLo) wines over the last decade that has been primarily driven by trends for moderation, wellness, and context-specific consumption rather than complete abstinence [1]. Although beer and ready-to-drink beverages have led this trend, wine has become increasingly popular with consumers because it allows them to retain cultural identity and sensory pleasure while lowering alcohol intake. A market forecast from the International Wine and Spirits Record (IWSR) suggests an overall volume growth of about 5% for NoLo beverages, with wine (+5%) progressing at a slower pace than beer (+6%) [2], reflecting the critical role that sensory expectations play in shaping wine consumption choices and the more mature state of NoLo beer production.

Recent EU regulation permits the production and labeling of dealcoholized wines (Reg. EU 2021/2117) [3]. Wines with less than 0.5% alcohol by volume are defined as “dealcoholized wine” and wine with alcohol strength above 0.5% and below the category minimum alcoholic strength are defined as “partially dealcoholized”. The term “NoLo” is therefore used in this paper in the context of this regulation.

The reduction in alcohol content in wine has become a key focus of both industry and academic research. Consequently, numerous physical separation technologies have been reviewed in recent comprehensive studies [4,5]. While these methods are effective in lowering ethanol content, they can simultaneously influence wine sensory profile by modifying volatile aroma compounds, phenolic composition, and color parameters [6,7,8,9]. These chemical modifications often lead to sensory changes, including lower aromatic intensity, poorer mouthfeel, and weakened expression of varietal character [6]. Such changes pose a major challenge for producers aiming to create NoLo wines that meet regulatory standards while preserving sensory authenticity. However, studies indicate that NoLo wines can remain acceptable to trained panels and regular consumers when alcohol reduction is moderate and key aroma and flavor characteristics are retained [10,11], with reductions of up to 20% alcohol. Moreover, the marketing of NoLo wines as harm-reduction (particularly dealcoholized wine) products may, in practice, contribute to the normalization of drinking, as their close visual and sensory resemblance to full-strength regular wines can encourage their consumption, particularly among younger consumers and those seeking to limit or avoid alcohol [12].

### 1.1. Research Scope

Consumer surveys indicate a gradual but increasing uptake of NoLo wines, driven by its sensory similarity to regular wine, prior experience, and value for money. An Australian study of 851 consumers found that purchase intention rose sharply with perceived taste similarity, especially among women and habitual mealtime wine drinkers [13]. Another Australian study likewise found high consumer awareness but hesitation to try NoLo wines. Tasting improved liking and purchase intent, yet shelf prices exceeded consumers stated limits, and willingness to pay sometimes declined after exposure [14]. Evidence from numerous national markets further highlights demographic and cultural variations, with younger and less frequent wine drinkers generally showing more openness [15,16]. Moreover, a Spanish study found increasing interest in dealcoholized wine driven by health and sustainability, but noted that limited availability, taste concerns, and low awareness still hinder wider adoption [17].

The NoLo wine market is growing more rapidly in the United Kingdom, Australia, the United States of America, and northern Europe. In Italy, the market is still emerging, with higher demand in Lombardy and Emilia Romagna, where NoLo wines are increasingly used as lighter aperitifs [18]. A survey conducted in the Apulia region found that willingness to purchase was primarily concentrated among younger and less frequent wine drinkers, while many others questioned its value and expected prices to be lower in proportion to the level of alcohol removed [19]. This limited regional evidence leaves a gap in understanding consumer attitudes toward dealcoholized wine across Italy. Evidence remains limited in traditional wine regions, particularly in Italy. Trentino-Alto Adige is a region with a strong wine culture, a premium-quality reputation (more than 94% of the wine stock is under PDO or PGI [20]) and provides a meaningful context for exploring consumer responses to NoLo wine. An examination of the NoLo market in this context could help clarify how awareness, perceptions, and expectations, along with price sensitivity, quality cues, and sensory factors, influence initial consumer acceptance of such products.

In summary, NoLo wine is an emerging product category, and consumer responses vary depending on cultural and market-dependent factors. Studies have covered consumer attitudes to NoLo wine in several different markets, but gaps remain. Attitudes in traditional premium winegrowing regions in northern Italy are still unknown and may differ both from non-winegrowing regions and from premium winegrowing regions in southern Italy.

Although this study does not apply a formal behavioral model, familiarity, social acceptance, and willingness to recommend are common topics in consumer research [21]. These links provide a conceptual basis for selecting the variables used in the survey. By relying on a structured survey, the present study builds on prior consumer survey research to investigate how conventional wine consumption patterns, familiarity with NoLo beverages, and sociodemographic factors shape perceptions and acceptance of NoLo wines in Trentino-Alto Adige, where NoLo wines remain relatively novel and are not yet widely adopted. In doing so, it provides new insights into adoption barriers and motivators. It also highlights opportunities for wineries and marketers to strengthen [22] communication strategies, improve market positioning, and build consumer trust in NoLo wine as a credible complement to conventional products.

### 1.2. Hypotheses Development

Prior studies on NoLo wines indicate that familiarity and acceptance differ based on drinking frequency and consumer involvement [21]. Consumer involvement theory suggests that people who engage more often with a product category pay closer attention to it, develop greater knowledge, cognitive structures, and familiarity with repeated exposure [23,24]. Market surveys reveal significant overlap between regular and NoLo drinkers. For instance, the IWSR reports an increasing proportion of drinkers, particularly Millennials, who consume both full-strength and no-alcohol options. This indicates greater exposure and familiarity among frequent wine consumers [25].

In contrast, Innovation-resistance literature shows that consumers reject products that deviate from traditions, identity cues, or expected product attributes [22]. According to Diffusion of Innovations [26], consumers deeply embedded in a category often act as innovation-resistant “late adopters” when the innovation disrupts valued attributes. Expectation–value and disconfirmation theories suggest that consumers who are very familiar with a product category tend to react more negatively when a product does not match what they expect [27].

Additionally, food (and technology) neophobia predicts lower willingness to accept products perceived as atypical or technologically altered, especially among consumers with strong attachment to traditional product characteristics [28]. According to the Theory of Planned Behavior, subjective norms or the perceived social expectations within a relevant group, influence individuals’ intentions and behaviors. If frequent wine drinkers perceive that recommending NoLo wines is inconsistent with the norms or identity of their wine-involved social group, this social pressure may discourage them from recommending such products [29].

Italy in general and Trentino-Alto Adige in particular have a long tradition in the wine industry [30]. Consumers who frequently drink wine are also more likely to try NoLo wines [31]. While NoLo wines are emerging as a socially influenced consumption trend among younger Italian consumers, broader social acceptance, especially among traditional wine drinkers, may still be limited in contexts with strong wine heritage [32]. Together, these theoretical perspectives explain why frequent wine drinkers may simultaneously (a) be more familiar with NoLo wines due to higher involvement and exposure yet (b) exhibit lower acceptance and lower recommendation intentions because NoLo wines conflict with established expectations, norms, and valued category attributes. Based on this (a, b) reasoning, the following hypotheses are proposed:

**H1.**  *Frequent wine drinkers [Q1] will be more familiar [Q8] with NoLo wines.*

**H2.**  *Frequent wine drinkers [Q1] will have lower social acceptance [Q16] of, and willingness to recommend [Q21], NoLo wines.*

Frequent consumers of alcohol-free beverages tend to exhibit greater openness toward, and stronger advocacy for, no-alcohol alternatives in general. Studies show that when alcohol-free options are clearly available, consumers are more likely to choose them, indicating positive attitudes among those who already drink such products [33]. Other consumer-based studies have also shown positive attitudes toward no-alcohol beer among those who already consume alcohol-free drinks [14,34]. This further highlights the potential acceptance of NoLo wines within such groups. Therefore, we propose the following hypotheses:

**H3.**  *Alcohol-free drinkers differ from alcohol free non-drinkers [Q2] in trying NoLo wines [Q9].*

**H4.**  *Alcohol-free drinkers differ from alcohol free non-drinkers [Q2] regarding the social acceptance [Q16] and willingness to recommend [Q21] NoLo wines.*

NoLo wines are often perceived as having lower sensory quality than regular wines, particularly in terms of taste, and this has consistently been identified as the primary barrier to acceptance [13,35,36]. Consumer studies show that demographic characteristics play a central role in shaping attitudes and behaviors toward NoLo wines. Evidence from choice modeling studies demonstrates that younger and less frequent drinkers are more open to dealcoholized wines than older and more involved consumers [19]. Although gender effects are context-dependent, studies have shown that women, particularly those young to middle-aged, are often more receptive to lower-alcohol wine and more likely to purchase it [37]. Therefore, the following hypothesis is proposed:

**H5.**  *There are differences across demographic variables (e.g., age and gender) in NoLo wine tasting experience [Q9], familiarity [Q8], purchase frequency [Q13], social acceptance [Q16], and willingness to recommend [Q21].*

## 2. Methodology

### 2.1. Survey Design

A descriptive, cross-sectional survey was designed to capture wine-consumption habits, familiarity with and perceptions of NoLo wine, intended consumption contexts, motivators and barriers to tasting experience, and socio-demographic characteristics. The questionnaires were developed through a combined approach. Several items were adapted and modified from previous consumer studies on NoLo wines [14,19,38], while additional questions were generated to capture context-specific factors considered relevant for this study. The full survey questionnaire used in this study is provided in the Supplementary Material Questionnaire.

The survey was organized in three sections to capture complementary dimensions of consumer behavior and perceptions. The first section, which covered wine consumption behavior, assessed general drinking habits, frequency of wine consumption, preferred wine styles, and typical spending patterns. The second section, which covered perceptions of NoLo wine, began with a brief definition of dealcoholized and partially dealcoholized wine consistent with EU Regulation 2021/2117. This section examined familiarity and prior tasting experience, expectations regarding quality and price, the importance of sensory attributes (taste, aroma, mouthfeel, appearance), perceived social acceptance, and likely consumption occasions. The final section collected socio-demographic information, including age, gender, occupation, region, and income. The questionnaire consisted of closed-ended items, primarily single-choice and multiple-choice formats, supplemented by Likert-type scales to assess importance and perceptions.

### 2.2. Ethical Approval and Consent

As the study involved the collection of personal data (e.g., demographic information, self-reported alcohol consumption habits), ethical approval was obtained in advance from the Research Ethics Committee of the Free University of Bozen-Bolzano (24 July 2024, Prot. Nr. UNIBZ_ AGR_NoLoWine_2024_23). Informed consent was integrated into the online survey. Before beginning the questionnaire, respondents were presented with an information sheet outlining the study’s purpose, anonymity assurances, and data use under GDPR compliance. Only participants who provided explicit consent by clicking “Yes” were able to proceed to the questionnaires.

### 2.3. Sample and Data Collection

The survey was conducted in winter 2024–2025 in Trentino-Alto Adige (Italy) using the online platform SurveyMonkey (San Mateo, CA, USA). The questionnaire was administered in English, and the detailed list of questionnaire items is provided in the Supplementary File for reference. The survey was advertised using both digital and physical approaches to maximize accessibility. A QR code linking to the survey was printed on posters and placed in public places, allowing interested individuals to access the survey directly via their smartphones. Participation was voluntary, and no financial incentives were provided. Of the 488 individuals who initiated the survey, after excluding non-consented and partial submissions, the final analytic sample comprised *n* = 344 respondents (completion rate 70.5%). The sample was 59.3% female, with participation concentrated in the 18–24 (50.0%) and 25–34 (32.9%) age bands, and a large share of students (67.7%). Full demographic distributions are reported in Table 1.

The survey was open to adults of all ages; the final respondent pool was largely composed of younger adults aged 18–35, reflecting the demographic that voluntarily participated. This emergent composition was not the result of intentional targeting, but it provides meaningful insights into the segment most engaged with novel wine products. Young adults are widely reported to be early adopters of NoLo beverages, and their consumption patterns can offer valuable indications of emerging trends in the NoLo market. Although this limits generalizability to the broader adult population in Trentino-Alto Adige, the data remains relevant for understanding the preferences and behaviors of a key consumer group driving the growth of NoLo wines.

### 2.4. Data Processing and Analysis

The analysis consisted of three methodological steps that allowed for the exploration of the NoLo wine market in Italy, in particular in the region of Trentino-Alto Adige.

Nominal variables were visualized using graphical plots such as bar plots with Microsoft Excel version 2510 (Microsoft Corporation, Redmond, WA, USA). This enabled comparisons between NoLo wines attributes, and highlighted those that better captured consumers’ attitudes, opinions, and perceptions.

Inferential statistical tests were applied to examine the study hypotheses using SPSS Statistics 29.0 (IBM, Armonk, NY, USA). Non-parametric methods were applied due to the ordinal and categorical nature of the survey data. All single-response items were measured on the scales indicated, and higher values represent stronger levels of the measured variable. For analysis, items presented in reverse order in the questionnaire (e.g., scales displayed from “extremely familiar” to “not at all familiar”) were recoded so that 1 always represents the lowest level and the highest value represents the strongest level of the response. Binary variables are coded as 0 = No and 1 = Yes, see Table S1.

H1 and H2 were tested using Spearman’s (1901) rho correlation, whose use is appropriate for assessing associations between ordinal variables [39]. H3 was examined using Chi-square tests of independence to test the association between the variables, whereas H4 and H5 were tested using Kruskal–Wallis tests to determine the difference between the age and gender groups for ordinal variables. In the case of NoLo wine tasting experience (Q9), which is a binary variable, Chi-square tests were used to examine its association with age and gender.

Finally, the variable “purchase frequency of NoLo wines” [Q13], which is originally of an ordinal nature, is rearranged into the binary variable B13, so that 0 stands for “never” and 1 for “at least sometimes”, the latter category encompassing the responses “rarely”, “sometimes”, “usually” and “always”. This decision was made to make variable Q13 operationalizable in econometric terms, in that—as will emerge from the descriptive statistics—the vast majority of the respondents never consume NoLo wines, while the categories “usually” and “always” count only a few answers. This does not allow for the correct estimation of models such as ordered logit and ordered probit, thus requiring a switch to the more manageable counterparts represented by logit and probit models. Indeed, the latter are actually estimated to investigate the relationship between the rearranged “purchase frequency of NoLo wines” [B13] and several other determinants.

Formally, given the index function $x^{\prime }\beta$, where x is a K × 1 vector of regressors and β is a vector of unknown parameters, the conditional probability p is given by

(1) $$p=P\left(B13=1|x\right)=F\left(x^{\prime }\beta \right)$$

where F(·) is a cumulative distribution function defined on the interval (−∞,∞), which ensures that p∈[0, 1] and P **(·)** denotes the conditional probability operator. More specifically, Equation (1) refers to a logit model when F(·) is the cumulative distribution function of the logistic distribution, while it refers to the probit model when F(·) is the standard normal cumulative distribution function. Parameters β are estimated via maximum likelihood. The decision to exploit the features of two distinct yet similar models to describe the purchase frequency of NoLo wines is rooted in the essentially exploratory nature of this work. Indeed, the use of one model served to evaluate the robustness and, thus, to corroborate the results of the other, and vice versa. All econometric calculations have been carried out using STATA 17 [40].

Given the number of bivariate tests performed, the risk of inflated Type I error should be considered, although no formal correction for multiple comparisons was applied.

## 3. Result and Discussion

### 3.1. Descriptive Findings

The sample was relatively diverse in terms of gender, with 59.3% identifying as female and 39.0% as male, while a very small proportion identified as non-binary or preferred not to disclose their gender. Age distribution was heavily concentrated in the younger groups, with half of the respondents between 18 and 24 years and nearly one-third between 25 and 34 years. The representation of older age groups decreased steadily, with only one participant above the age of 65.

The demographic profile of the sample consisted mostly of younger adults and students, with a higher proportion of female respondents. Because of this, the findings should be interpreted with some caution when discussing consumers in a premium wine region like Trentino-Alto Adige. The results mainly reflect the views of younger wine consumers rather than the full regional population, which may limit how broadly the findings can be applied. However, younger consumers play a growing role in the NoLo market, so their views highlight the trend in the NoLo wine market.

#### 3.1.1. General Wine Consumption Behavior

Table 2 summarizes the general wine consumption habits of the 344 respondents. Most participants reported consuming wine on a weekly (29.1%) or monthly (24.1%) basis, while only a small proportion (3.5%) drank wine daily. Notably, nearly 15% of respondents indicated that they never consume wine among our sample, reflecting the growing presence of occasional and abstaining drinkers in Italy, consistent with recent reports of declining wine consumption in traditional wine-producing countries due to lifestyle changes and health-oriented choices [41,42]. When asked about general alcohol-free beverage consumption, responses were almost evenly split, with 50.3% reporting that they typically consume alcohol-free drinks. This finding reflects broader shifts in beverage preferences, where NoLo options are increasingly integrated into consumer repertoires rather than being associated solely with abstainers [43].

In terms of wine styles, red wine (57.3%) and dessert wine (57.0%) were the most preferred categories, followed closely by white wine (55.5%) and sparkling wine (37.5%), whereas only 17.7% indicated a preference for rosé. Interestingly, 8.4% of respondents stated that they had no preference, which may reflect openness toward diverse wine styles, while 1.7% selected “other,” suggesting niche or alternative preferences not captured by conventional categories. Compared with previous Italian consumer studies, which have consistently shown red wine as dominant, but with rising interest in sparkling wines [44,45], the strong showing of dessert wines in our sample may reflect the personal liking and local cultural traditions specific to the Trentino-Alto Adige region. Regarding expenditure, most respondents reported spending €10–20 per bottle (45.6%), followed by those spending less than €10 (38.4%). Only a small fraction spent above €20, with 12.2% in the €20–30 range, 2.6% in the €30–40 range, and just 1.2% above €40. This distribution suggests a predominantly value-conscious market segment, consistent with previous research indicating that Italian consumers generally concentrate their spending in the mid-price range and perceive higher-priced bottles as occasional or premium purchases [46].

**Figure 1:**
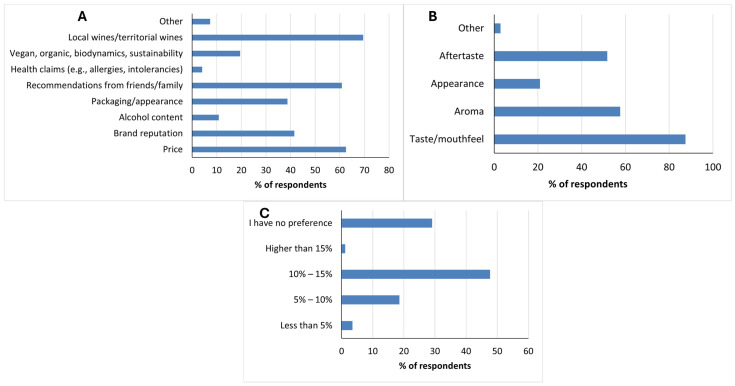
Illustrates responses on (**A**) factors influencing wine purchase decisions (multiple responses permitted) [Q5], (**B**) sensory attributes considered most important when evaluating wine (multiple responses permitted) [Q6], and (**C**) preferred alcohol content levels in wine [Q7].

As shown in Figure 1A, consumers wine purchasing decisions were primarily driven by local origin (69.5%), price (62.5%), and recommendations from friends or family (60.8%), while factors such as brand reputation (41.5%), packaging/appearance (38.7%), and especially alcohol content (10.8%) and health claims (4.1%) were less decisive. These results point to regional identity and price as the main factors guiding consumer choices in the Trentino-Alto Adige wine market. Palmieri and Perito [47] demonstrated that Italian consumers associate local origin with authenticity and quality, while Gow et al. [48] confirmed that price continues to heavily condition willingness to pay for sustainable or innovative wines. In addition, our respondents also prioritized price, recommendations, and local origin over health-related cues. This is an agreement with Costanigro et al. [49], who found that consumers were willing to pay more for wines indicating organic and without sulfites, but ultimately based their decisions on price and quality. Nevertheless, the 19% interest in organic and sustainable wines, although modest, indicates a growing consideration for these attributes within conventional wine choices among respondents. In terms of sensory drivers (Figure 1B), taste and mouthfeel were rated most important (87.5%), followed by aroma (57.6%) and aftertaste (51.7%), while appearance was comparatively minor (20.9%). A similar result was obtained by Corduas et al. [50], who reported aroma and taste as the most influential attributes among Italian consumers, whereas color was given less importance. This shows that consumers’ sensory expectations are anchored in flavor authenticity rather than external presentation.

Regarding preferred alcohol strength, nearly half of respondents (47.7%) selected 10–15%, reflecting a strong orientation toward the traditional alcohol levels found in many Italian reds and structured whites. A further 29.1% expressed no preference, while relatively few favored 5–10% (18.6%) or <5% (3.5%), as shown in Figure 1C. Over half of the respondents either favored lower-strength wines or indicated no specific preference, suggesting that consumers may be open to alternatives if these wines deliver a sensory experience comparable to that of traditional wines. These findings align with trends observed in a broader international sample: a recent survey across France, Germany, Italy, and the United States found that preferences for low-alcohol wine increased slightly from 36% in 2022 to 40% in 2024, though the majority still preferred traditional strength or indicated no preference [15]. Taken together, these findings illustrate that respondents evaluate wine primarily through cultural authenticity, sensory quality, and affordability, with health- and alcohol-related cues playing a minor role in selecting conventional wines.

#### 3.1.2. Consumer Awareness and Perceptions Toward NoLo Wines

**Figure 2:**
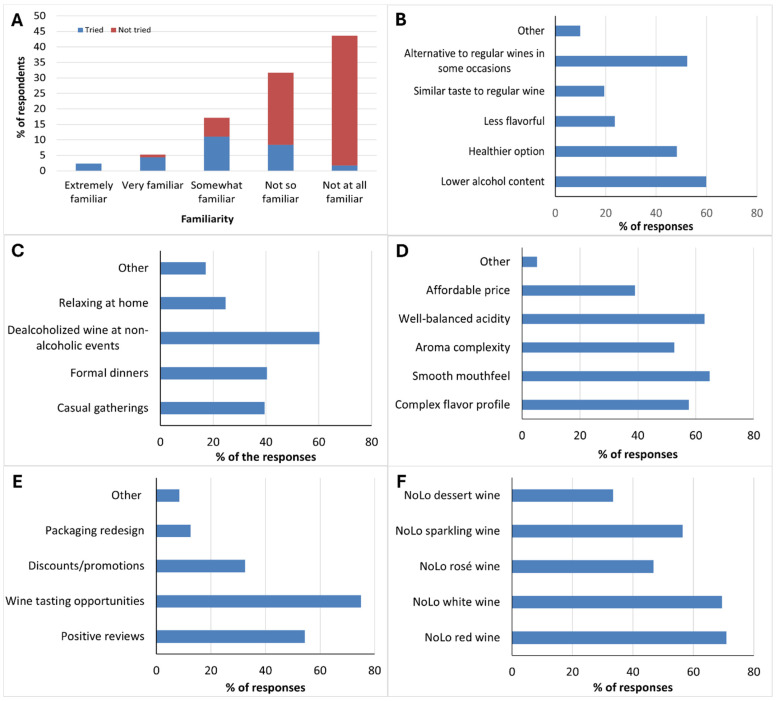
(**A**) Familiarity with NoLo wines and tasting experience. Bars show the share of all respondents in each familiarity category; colored segments indicate the percentage within each category who reported having tried or not tried NoLo wine [Q8, Q9]. (**B**) Consumer perceptions of NoLo wine: Proportion of respondents associating given attributes (multiple responses permitted) [Q11]. (**C**) Preferred contexts for consuming NoLo wine: Situational use patterns among respondents (multiple responses permitted) [Q12]. (**D**) Consumer expectations of high quality in NoLo wine: Proportion rating each attribute as important to their perception of high quality (multiple responses permitted) [Q17]. (**E**) Encouraging factors to try NoLo wine among respondents (multiple responses permitted) [Q19]. (**F**) Types of NoLo wine respondents would be willing to try: proportions selecting each style (multiple responses permitted) [Q20].

Familiarity with NoLo wine was assessed on a five-point self-report scale ranging from extremely familiar to not at all familiar. The overall familiarity was found to be low, since only 2.3% of respondents described themselves as extremely familiar and 5.2% as very familiar, while 17.2% reported being somewhat familiar. By contrast, the majority indicated little awareness, with 31.7% selecting not so familiar and 43.6% not at all familiar Figure 2A. This distribution shows that nearly three-quarters of respondents had limited or no familiarity with NoLo wines. Overall, 27.9% of respondents reported having tried a NoLo wine, whereas 72.1% indicated no prior experience with such products.

The combined result of familiarity and tasting experience was largely concentrated among respondents with higher familiarity, whereas those reporting little or no familiarity overwhelmingly comprise the group who have not tried NoLo wines, as shown in Figure 2A. These findings suggest that NoLo wines remain relatively unknown to consumers in Trentino-Alto Adige, with both familiarity and tasting experience rates being low. Similar results have been observed in earlier Italian research, where awareness of dealcoholized wine was low, and purchase intentions were limited unless accompanied by lower prices [19]. In contrast, an Australian study reported considerably higher levels of familiarity with NoLo beverages compared to the findings presented here, although awareness of NoLo wine still lagged behind that of non-alcoholic beer [14]. Recent cross-national surveys also confirm that the consumption experience for NoLo wines is consistently below that for beer and ready-to-drink products, which typically lead to the growth in the NoLo category [2]. The contrast suggests that in regions with a strong traditional wine culture, NoLo wines have yet to achieve meaningful consumer penetration. The close link between familiarity and tasting experience in our data further indicates that increasing opportunities for tasting and raising product visibility could be key to building awareness and normalizing consumption.

Figure 2B–F present results from multiple-response questions, where respondents could select more than one option. Figure 2B shows the perceptions that respondents associated with NoLo wine. The majority, 59.9%, identified it primarily as a reduced-alcohol product, while 48.3% connected it with the perception of health benefits. Similarly, over half of the respondents (52.3%), viewed NoLo wine as a potential alternative to regular wine in certain situations. Additionally, fewer respondents considered it less flavorful (23.6%) or believed it could provide a taste like conventional wine (19.5%). These motivations align with broader evidence suggesting that consumers are increasingly seeking reduced-alcohol options as part of health-oriented or moderation-driven consumption [16,37]. Regarding contexts of consumption reported in our study (Figure 2C), 60.2% considered NoLo wine most appropriate for non-alcoholic events, followed by 40.4% for formal dinners and 39.5% for casual gatherings, whereas only 24.7% selected relaxing at home. These results indicate that NoLo wine is mainly viewed as suitable for social occasions rather than for private, everyday drinking. This aligns with Johnson et al. [51], who found that nonalcoholic wine is often chosen in social settings because it allows people to take part in drinking rituals without alcohol. Recent research also underscores that NoLo consumption is highly context-dependent, with drinking occasion, time of day, and social setting emerging as key factors guiding when such products are deemed suitable [52].

Figure 2D illustrates respondents’ expectations for what constitutes a high-quality NoLo wine. The results show a clear emphasis on sensory attributes, with smooth mouthfeel, well-balanced acidity, complex flavor profile, and aroma complexity all ranking highly over 50%. In contrast, economic considerations such as price were less frequently prioritized, mentioned by only 39% of respondents. The prioritization of sensory attributes is consistent with findings by Bucher et al. [53], who demonstrated that improvements in the sensory quality of low-alcohol wines significantly increased consumer acceptance, reinforcing the role of sensory performance as a key determinant of perceived quality. These findings suggest that, for consumers in Trentino-Alto Adige, quality in NoLo wine is defined primarily through its sensory performance rather than price, underscoring the expectation that such products should match the standards of conventional wine. This emphasis on sensory quality is in line with Waehning and Wells [52], who highlighted sensory cues as key drivers of NoLo wine acceptance, although they also noted that the overall evidence remains limited and inconsistent.

Figure 2E presents the factors that would encourage respondents to try NoLo wines. The most frequently selected motivator was wine tasting opportunities, chosen by 75% of respondents, followed by positive reviews (54.4%). Discounts or promotions were less persuasive, identified by 32.6%, while packaging redesign was selected by only 12.5%. The strong preference for tastings (75%) and positive reviews (54%) observed reflects widespread evidence that consumers rely heavily on sensory experience and social validation when considering NoLo wines. As reported by Lockshin and Corsi [54], tasting events are critical in driving wine purchase behavior because they allow consumers to evaluate quality directly. Similarly, research on low alcohol wines emphasizes that when the sensory quality is compelling, consumer acceptance increases significantly [37]. However, discounts or promotions were less influential in our study, suggesting that within this emerging category, quality cues carry more weight than cost in motivating willingness to try these wines. This contrasts with the findings of Stasi et al. [19], who reported expected price reductions for dealcoholized wine in their survey. It must be noted, however, that the average per capita income in Puglia (17,000 Euro, 2022) is lower than Trentino Alto Adige (24,000 Euro, 2022) [55]. As shown in Figure 2F, the distribution of willingness to try NoLo wine types reveals a clear preference for red and white NoLo wine, followed by sparkling and rosé. Although 57% of the respondents chose conventional dessert wine as one of their preferred wines (similarly to white and red conventional wines), NoLo dessert wine was the least favored style, chosen by only 33.4% of respondents. That NoLo dessert wine was not seen favorably by respondents may reflect its niche role in meals or the uncommon presence of NoLo dessert-style products in everyday contexts.

### 3.2. Hypothesis Testing

Spearman’s rank-order correlation was examined to test the proposed hypotheses *H1* and *H2*. As predicted in hypothesis *H1*, wine drinking frequency was positively and significantly associated with familiarity with NoLo wines, *ρ*(344) = 0.166, *p* = 0.002. This indicates that individuals who consume wine more frequently tend to report higher levels of familiarity with dealcoholized alternatives, and therefore *H1* is supported. In contrast, *H2* received only partial support. While drinking frequency was not significantly related to perceptions of social acceptance, *ρ*(344) = 0.018 and *p* = 0.736, a significant negative association emerged between drinking frequency and willingness to recommend, *ρ*(344) = −0.212, and *p* < 0.001, suggesting that frequent wine drinkers are less inclined to recommend NoLo wines compared to those who drink less often.

The significant association between frequent wine drinkers and greater NoLo wine familiarity is consistent with the recent evidence showing that familiarity and prior experience increase acceptance of novel food and beverage products. In wine research, higher involvement is linked to greater knowledge, offering an explanatory pathway for why regular drinkers in our sample are more familiar with NoLo wines. Since limited awareness is a known barrier to NoLo adoption, consumers who are more engaged in wine culture are also more likely to have encountered these products, consistent with our findings [21,56,57,58]. Although *H2* predicted that frequent drinkers would view NoLo wines as less socially acceptable, no significant association was found. This suggests that perceptions of social acceptability may not depend on drinking frequency but may instead reflect broader cultural or health-related values. Recent research highlights that NoLo acceptance is often driven by health and functional motivations, while situational or social values play a weaker role, which may explain the absence of an effect in our data [21]. This indicates that social acceptance of NoLo wines may be relatively consistent across consumer groups, regardless of wine drinking habits. The lower willingness of frequent wine drinkers to recommend NoLo wines likely reflects quality and sensory concerns, since dealcoholization often reduces body and balance and increases bitterness. This causes experienced consumers to rate them less favorably than standard wines, as has been reported in previous studies [4,59].

To test *H3*, a Chi-square test of independence was conducted to examine drinkers and non-drinkers of alcohol-free drinks in relation to the tasting experience of Nolo wines. The association between the two variables was not statistically significant (Table S2). This indicates that the likelihood of having tried NoLo wines does not significantly differ between those who consume alcohol-free drinks and those who do not. Thus, *H3* was not supported.

The Kruskal–Wallis test was used to test *H4* (see Table 3). The results showed a significant difference in social acceptance among groups, *H*(1) = 4.447, *p* < 0.05, and a highly significant difference in willingness to recommend, *H*(1) = 23.121, *p* < 0.01, supporting *H4*. These findings indicate that individuals who consume alcohol-free drinks tend to show higher social acceptance and a greater willingness to recommend NoLo wines compared to those who do not consume alcohol-free beverages.

**Figure 3:**
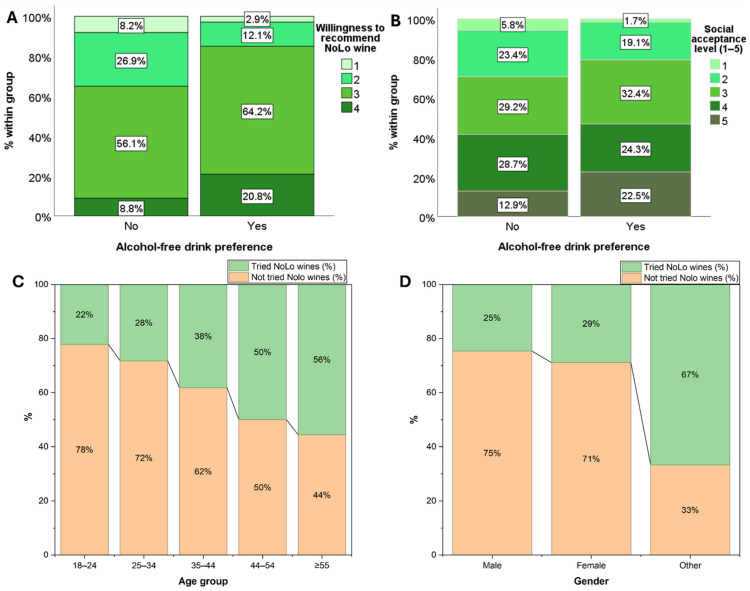
Distribution of (**A**) social acceptance levels (1 = not at all acceptable–5 = Totally acceptable) and (**B**) willingness to recommend NoLo wine (1 = definitely would not–4 = definitely would) among respondents with and without an alcohol-free drink preference. Each bar represents 100% of the respective group, subdivided by response categories. Respondents with an alcohol-free drink preference show a greater share in the higher response levels compared to those without such preferences (Note for fig. A and B X axis: No, do not drink alcohol free drinks; Yes, alcohol free drinkers). (**C**) Percentage of respondents who have tried and not tried NoLo wine across age groups. Tasting experience increases progressively with age, with the highest proportion of “tried” responses observed among older participants. (**D**) percentage of respondents who have tried and not tried dealcoholized wine across gender groups.

The response distribution can explain the significant difference observed for social acceptance (*H4*). Alcohol-free drink consumers were over-represented in the highest acceptance category (63.9%), indicating stronger approval of NoLo wines, whereas non-consumers were predominantly concentrated in the lowest acceptance category (76.9%), reflecting limited social acceptance among this group. Similarly, among those who would definitely recommend NoLo wine, 70.6% were alcohol-free drink consumers (vs. 29.4% non-consumers), whereas non-consumers predominated among those unlikely to recommend (“definitely would not” = 73.7%; “probably would not” = 68.7%) (Table S3). Furthermore, Figure 3 shows the distribution of social acceptance (A) and willingness to recommend dealcoholized wine (B) across respondents with and without an alcohol-free drink preference, complementing the reported results. Shaw et al. [21] observed that nearly 39% of respondents had tried non-alcoholic wine and 44% had tried low-alcohol wine, highlighting greater openness among those who already consume NoLo wine. In contrast, our findings extended this pattern by showing that even general alcohol-free drink consumption (beyond wine alone) is associated with higher acceptance and willingness to recommend NoLo wines. Similarly, Lamorte and Agnoli [60] further demonstrated that positive attitudes and perceived social norms significantly increase intentions to consume dealcoholized wines, which in our study is seen in the form of significant social acceptance among alcohol-free drinkers. However, the null association observed for *H3* may be explained by the relatively limited availability of NoLo wines in the market compared with other categories, such as NoLo beers, which are already widely distributed and familiar to consumers. This lack of exposure likely reduces opportunities for tasting experience, even among alcohol-free drink consumers [61].

The higher acceptance observed among alcohol-free drink consumers in our study might reflect broader health and moderation-oriented motivations. These are also evident in the categorical responses shown in Figure 2B, where respondents expressed strong interest in NoLo wines as a healthier option, i.e., an alternative to regular wine due to their lower alcohol content. This reasoning is further supported by market reports showing the rise of substituters, consumers who flexibly alternate between full-strength and NoLo beverages depending on the occasion, indicating that NoLo products are becoming integrated into mainstream drinking patterns and increasingly normalized in social contexts [43].

Chi-square tests revealed a significant association between age group and NoLo wine tasting experience, *χ*^2^(4) = 12.003, *p* < 0.05, indicating that the likelihood of having tried NoLo wines differed across age groups. A weak, non-significant association was observed between gender and tasting experience, *χ*^2^(2) = 5.301, *p* < 0.1, suggesting that men and women differed slightly in their reported trial of NoLo wines (see Table 4). As reported in Table S4 and represented graphically in Figure 3C, the proportion of respondents who had tried NoLo wines increased with age, ranging from 22.1% in the youngest group to 55.6% among the oldest. When examined by gender (Figure 3D), differences were less pronounced: a slightly higher proportion of women respondents (28.9%) than men (24.6%) reported having tried NoLo wine, while those identifying as “other” showed a comparatively higher tasting experience rate (66.7%). However, this result is based on a small sample size.

For age, a significant difference was observed in NoLo wine familiarity, *H*(4) = 10.850, *p* < 0.05, indicating that familiarity with NoLo wines varied among age groups. However, no significant differences were found for purchase frequency, social acceptance, and willingness to recommend. For gender, significant differences were found in NoLo wine familiarity, *H*(2) = 8.343, *p* < 0.05, and highly significant differences were observed for both social acceptance, *H*(2) = 12.794, *p* < 0.01, and willingness to recommend, *H*(2) = 17.803, *p* < 0.01. However, no significant gender difference emerged for purchase frequency (see Table 5).

**Figure 4:**
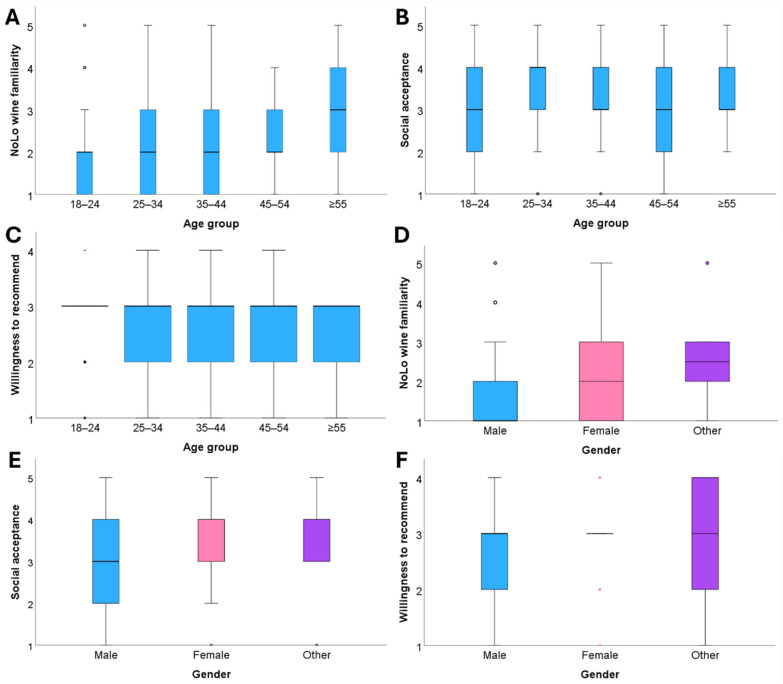
Boxplots showing age and gender related differences (H5) in NoLo wine familiarity, social acceptance, and willingness to recommend. (**A**) Boxplot showing age related differences in NoLo wine familiarity. (**B**) Boxplot showing age-related differences in perceived social acceptance of NoLo wines. (**C**) Boxplot showing age-related differences in willingness to recommend NoLo wines. (**D**) Boxplot illustrating gender-related differences in NoLo wine familiarity. (**E**) Boxplot showing gender-related differences in social acceptance of NoLo wines. (**F**) Boxplot illustrating gender-related differences in willingness to recommend NoLo wines. Age and gender effects were assessed using Kruskal–Wallis tests. Familiarity increased with age, while female respondents reported higher familiarity, greater social acceptance, and stronger willingness to recommend NoLo wines. Each boxplot displays the median (center line), interquartile range (IQR), and whiskers extending to 1.5 × IQR; circles or asterisks denote outliers. Higher scores correspond to stronger familiarity, social acceptance, and willingness to recommend NoLo wines.

The boxplot visualizations (Figure 4A–F) illustrate distributional differences across age and gender. Familiarity with NoLo wines differed notably across age groups, with respondents aged ≥55 showing the highest median familiarity scores (Figure 4A). In contrast, the boxplots for purchase frequency and social acceptance (Figure 4B,C) revealed broadly similar median values across age categories, confirming the absence of significant age-related trends. These findings suggest that although older consumers are more likely to have tried and to be familiar with NoLo wines, their attitudes and purchasing behaviors remain largely comparable to those of younger respondents. Across genders, median values (Figure 4D,F) were consistently higher for female respondents, reflecting stronger familiarity, social acceptance, and willingness to recommend NoLo wines. Although respondents identifying as “other” also reported relatively high median values, the sample size of this group was comparatively small. The combined evidence suggests that age primarily shapes experience-based engagement with NoLo wines (familiarity and tasting experience), whereas gender is more strongly associated with social acceptance and recommendation intentions, thereby providing partial support for *H5*.

Previous research often positions younger consumers as the primary audience for NoLo products, driven by health and moderation motives [51], yet reports barriers such as taste expectations, quality perception, and social norms that hinder actual adoption [21]. The higher tasting experience and familiarity observed among older respondents in our data likely reflects broader exposure to wine categories. This pattern can be understood based on Stasi et al. [19], who reported that dealcoholization often reduces perceived quality and that consumers, especially younger or less frequent drinkers, buy only when prices are lowered. This helps explain why younger consumers’ openness may not lead to actual consumption, while older consumers are more experienced and have been exposed to different wine options. However, in a recent UK study, Perman-Howe et al. [62] found greater NoLo consumption among younger adults, highlighting how market context shapes adoption. In Italy’s more traditional wine culture, limited availability may explain the opposite trend. New legislation [63] allowing wider production could increase accessibility among all age groups.

Women showed higher familiarity, social acceptance, and willingness to recommend, but no difference was observed in the NoLo wine tasting experience and purchase. This aligns with prior studies showing that health, moderation, and social-acceptability motives are more prominent among women drinkers, and these often enhance attitudinal evaluations even without higher behavioral uptake [37,53]. Previous Italian research reports that women, who consume wine less often and are more likely to prefer milder, lower-alcohol drinks, show favorable attitudes towards NoLo drinks without higher purchase frequency [64]. A recent review by Waehning and Wells [52] noted that factors such as availability, marketing, and information cues can influence NoLo consumption, but also stressed that existing evidence remains limited and inconsistent. While acceptance of NoLo wine in Trentino-Alto Adige remains low, the generally positive attitudes observed across age groups suggest strong potential for future growth. Although social acceptance currently sits at a moderate level, it could increase with improved product visibility, sensory quality, and opportunities for tasting, such as wine-tasting events, as discussed in the descriptive section (Figure 2E). Willingness to recommend, consistent across all ages, further indicates openness toward NoLo wines. Together, these patterns highlight an opportunity for Italian producers to strengthen consumer engagement by balancing tradition with innovation as the market evolves.

### 3.3. Logit and Probit Models

**Figure 5:**
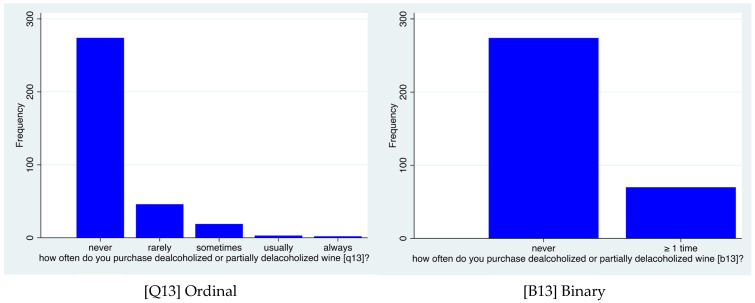
Comparison between the ordinal variable Q13 and its rearranged binary version B13.

As already stated in Section 2.4, the frequency distribution of the responses to question Q13 “purchase frequency of NoLo wines” does not allow for fully exploiting its ordinal content. This fact is captured by the left subplot of Figure 5 below, where it is possible to notice the relative abundance of “never” answers compared to all other categories of question Q13.

For this reason, the decision was taken to condense all responses other than “never” into the unique and more heterogeneous category “at least sometimes”, the full information being conveyed by the rearranged binary variable B13. This allows for the safe use logit and probit model to explore the determinants behind the frequency of NoLo wine purchases in the Trentino-Alto Adige region. It is worth stressing that the estimates of the logit model are not converted into odds ratios in order to keep potential sign consistencies with the estimates of the probit model. At this exploratory stage, robustness of results is preferred to ease of interpretation.

Table 6 investigates the relationship between the frequency of NoLo wine purchases.

In particular, seven variables (conventional wine drinking frequency, alcohol free drink status, spending habit, preferred alcohol level in wine, NoLo wine familiarity, NoLo wine tasting experience, NoLo wine taste importance) are used to describe the respondents’ behavior towards normal and NoLo wines, and three control variables (age, gender, income) to provide a socio-demographic context. In particular, the coefficients β  can be simply interpreted as “mathematically sophisticated” slope coefficients [65] linking one of the considered variables to NoLo wine purchase frequency, all other variables remaining constant.

First, the Likelihood Ratio (LR) Chi-Square is 185.63 for the logit and 183.69 for the probit model with 31 degrees of freedom each. Both are significant at the *p* < 0.01 level, thus signaling that at least one of the independent variables has a significant effect on NoLo wine purchase frequency. Both models share a pseudo-*R^2^* higher than 0.500.

It clearly emerges that, everything else being equal, categories concerning familiarity with NoLo wines have a positive and significant impact on purchase frequency when compared with people who are not familiar. This result is strong for the logit as well as for the probit model and supports prior findings that direct experience and exposure reduce uncertainty and increase purchase intention for NoLo products [13,59]. This finding also aligns with the evidence that acceptance increases with familiarity when sensory expectations are met. This has been reported in a study by Meillon et al. [59], which found that tasting and clear information improved liking for partially dealcoholized wine. Alongside familiarity, prior NoLo wine tasting experience also seems to share a very significant (*p* < 0.01) and positive relationship with purchase frequency in both models. This relationship is supported by Baiano [34], who reported that consumers with prior experience of non-alcoholic beverages (e.g., beer) demonstrated higher willingness to pay and stronger intentions to repurchase. Their results support our interpretation that prior tasting experience reduces perceived risk and fosters repeat purchasing, which may explain the strong positive association between prior tasting experience and purchase frequency observed in both models. With respect to the importance of the preferred alcohol level in wines on purchase frequency, when compared to the “no-preference” category, both models signal that alcohol contents between 5% and 10% have a positive effect on purchase frequency, while alcohol contents beyond 10% negatively affect the purchase frequency of NoLo wines. This could be explained by the results of earlier research, which showed that mid-to-low alcohol wines are generally as well-liked as standard wines during tasting [53], and that consumers motivated by moderation or health tend to choose lower-alcohol options [16,52]. The negative association between consumer liking, >10% ABV, and purchase frequency could be due to their higher sensory expectation and perceiving less sensory authenticity in NoLo wines widely reported in various studies [32]. However, neither the logit nor the probit models suggest a significant effect of a low alcohol content (<5%) on the frequency of NoLo wine purchases.

Other variables present relatively more heterogeneous results when shifting from the logit to the probit frameworks. As an example, NoLo drink preference has a slightly significant and positive connection with purchase frequency in the logit setting, while it becomes non-significant when dealing with the probit setting. Given the exploratory nature of this study and the still-developing NoLo wine market, such variability between model frameworks is not unexpected and underscores the importance of continued investigation with larger and more diversified samples to confirm these preliminary tendencies.

In general, it is worth mentioning that variables such as the frequency of traditional wine consumption, spending habits on traditional wines, and the relevance given to NoLo wine taste importance seem to play no role in influencing NoLo purchase frequency in our study. The lack of significance for taste importance likely reflects a ceiling effect, as most respondents already consider taste essential when evaluating any wine, reducing its ability to differentiate purchasing frequency. Overall, notwithstanding the non-generalizability of the results due to the socio-demographic characteristics of this strictly “local” sample, these results seem to suggest two interesting yet preliminary points. First, the frequency of NoLo wine purchases seems to be linked to a previous knowledge of NoLo wines. Second, the frequency of NoLo wine purchases does not seem to be influenced by its traditional counterpart. These considerations provide some potentially useful insights. Firstly, NoLo winemakers might benefit from organizing more events to allow potential customers to taste NoLo wines, which would introduce them to the product and reduce skepticism. Secondly, at least as far as Trentino-Alto Adige (a region historically characterized by a strong winemaking tradition) is concerned, producers should probably consider the NoLo wine market as relatively independent from that of normal wines. Given the exploratory nature of this work, however, these findings are far from being definitive and further research addressing samples with different socio-demographic characteristics in different geographical areas is needed to corroborate their stability.

## 4. Conclusions

This study examined how wine consumption habits, familiarity with NoLo beverages, and demographic factors influence perceptions of NoLo wines in a traditional wine region such as Trentino-Alto Adige (Italy). The participants’ profile was skewed toward a younger group (18–34, predominantly women) who are moderate wine consumers; typically spend €10–20 per bottle; and prioritize local origin, price, and peer recommendations when purchasing wine. Although overall familiarity and tasting experience with NoLo wines were low, respondents showed moderate openness toward the category. Respondents associated NoLo wines with lower alcohol content and perceived health benefits, perceiving it as suitable for social or non-alcoholic occasions. Tasting opportunities and positive reviews were identified as the most effective motivators.

Hypothesis testing indicated that frequent wine drinkers were more familiar with NoLo wines but less likely to recommend them, revealing a degree of resistance among traditional consumers. However, NoLo consumers showed significantly higher social acceptance and willingness to recommend. Differences by age and gender indicated greater familiarity among the older group and women respondents, but by gender, women emerged with higher social acceptance and willingness to recommend. Logit and probit models further identified familiarity as the main driver of purchase frequency, while traditional wine consumption showed no effect, suggesting that the NoLo category is developing as a relatively distinct segment within the wine market.

The findings uncover an emerging openness toward NoLo wines. Increasing familiarity through greater availability and consistent sensory quality could further strengthen consumer acceptance and integration of NoLo wines within Italy’s evolving wine culture. Although this study provides early insights into consumer perceptions of NoLo wines within the traditional wine culture of Trentino-Alto Adige, it is not without limitations. The analysis was region-specific, and results may therefore not be directly transferable to other areas with different wine traditions, market exposure, or cultural attitudes toward NoLo products. The sample was dominated by young and predominantly women respondents. This offers a valuable perspective on emerging consumer groups but potentially underrepresented older, more established wine drinkers who remain central to the conventional market. While this composition highlights the views of future consumers, it may limit the full demographic representativeness of the findings. Moreover, the modest sample size and exploratory nature of the statistical models suggest that further research with larger and more diverse samples is needed to strengthen external validity. In addition, there was some potential for self-selection bias, and people who are interested in wine or NoLo products might have been more likely to respond than those who are not. It also relied on self-reported consumption and attitudes. In addition, the cross-sectional design precludes causal inferences. Future studies could extend this work by comparing regions, incorporating longitudinal data, or examining how familiarity, sensory expectations, and marketing communication evolve as the NoLo category becomes more established. They could also include comparative studies across different Italian regions and a more explicit measurement of underlying motivations.

Furthermore, the study relied on single-item measures for constructs such as familiarity, social acceptance, and willingness to recommend. The reliability of these measures cannot be formally assessed. Future research could incorporate validated multi-item scales to improve measurement precision.

## Figures and Tables

**Table 1:** Socio-demographic characteristics of the sample (*n* = 344).

Characteristic	Category	% of Respondents
Gender	Male	39
	Female	59.3
	Non-binary/Other	0.3
	Prefer not to disclose	1.5
Age	18–24	50
	25–34	32.9
	35–44	9.9
	45–54	4.7
	55–64	2.3
	65+	0.3
Occupation	Student	67.7
	Teacher	4.9
	Business owner	0.9
	Employee	18.9
	Retired	0
	Other	7.6
Income	No income	29.7
	Under €15,000	32
	Between €15,000 and €29,999	21.8
	Between €30,000 and €49,999	10.2
	Between €50,000 and €74,999	4.4
	Between €75,000 and €99,999	0.9
	Over €100,000	1.2

**Table 2:** Frequency of wine consumption [Q1], alcohol-free drink habits [Q2], preferred wine types (multiple responses permitted) [Q3], and average spending per bottle [Q4] among survey respondents (*n* = 344).

Variable	Category	% of Respondents
Frequency of wine consumption	Daily	3.5
	Weekly	29.1
	Monthly	24.1
	Rarely	28.5
	Never	14.8
General alcohol-free drink behavior	Yes	50.3
	No	49.7
Preferred wine type	Red wine	57.3
	White wine	55.5
	Rosé wine	17.7
	Sparkling wine	37.5
	Dessert wine	57
	No preference	8.4
	Other	1.7
Average spending per bottle	<€10	38.4
	€10–€20	45.6
	€20–€30	12.2
	€30–€40	2.6
	More than €40	1.2

**Table 3:** Differences between alcohol-free drinkers and non-drinkers in social acceptance and willingness to recommend NoLo wines (Kruskal–Wallis test).

Dependent Variable (DV)	N. Categories (*K*)	Kruskal–Wallis (*H*)	Ties
Social acceptance	2	4.447 ** (1)	Yes
Willingness to recommend	2	23.121 *** (1)	Yes

Notes: Higher scores on both social acceptance and willingness to recommend were more common among consumers of alcohol-free drinkers, while non-consumers were more represented in the lower response categories. The underlying distributions for each group are provided in Supplementary Table S1. 1. Statistical significance assessed with respect to H(1); 2. ** for *p* < 0.05, *** for *p* < 0.01. Source: Authors’ calculations.

**Table 4:** Results of Chi-square analyses reporting the associations between demographic variables and NoLo wines tried status.

Dependent Variable (DV)	Independent Variable	Categories (*k*)	Chi-Squared *χ^2^* (df)
Tried NoLo wine	Age	5	12.003 ** (4)
Tried Nolo Wine	Gender	3	5.301 * (2)

Notes: 1. Statistical significance assessed with respect to $\chi^{2}(1)$; 2. * for *p* < 0.1, ** for *p* < 0.05. Source: Authors’ calculations.

**Table 5:** H5 results of nonparametric tests “Kruskal–Wallis” comparing Age-group (5 categories) and Gender (3 categories) differences in NoLo wine familiarity, purchase frequency, social acceptance, and willingness to recommend.

Age			
Dependent Variable (DV)	Categories (*k*)	Kruskal–Wallis *H* (df)	Ties
NoLo wine familiarity	5	10.850 ** (4)	Yes
NoLo wine purchase frequency	5	3.448 (4)	Yes
Social acceptance	5	3.873 (4)	Yes
Willingness to recommend	5	5.797 (4)	Yes
**Gender**			
**Dependent Variable (DV)**	**Categories (*k*)**	**Kruskal–Wallis *H* (df)**	**Ties**
NoLo wine familiarity	3	8.343 ** (2)	Yes
NoLo wine purchase frequency	3	2.789 (2)	Yes
Social acceptance	3	12.794 *** (2)	Yes
Willingness to recommend	3	17.803 *** (2)	Yes

Notes: 1. Statistical significance assessed with respect to H(1); 2. ** for *p* < 0.05, *** for *p* < 0.01. Source: Authors’ calculations.

**Table 6:** Logit and probit explaining the frequency of NoLo wine purchases.

Dependent Variable: [Q13] Frequency of NoLo Wine Purchase		
Model	Logit	Probit
[Q1] Traditional wine drinking frequency		
1. rare (base level)	–	–
2. from time to time	0.486 (0.730)	0.331 (0.390)
3. monthly	0.540 (0.738)	0.367 (0.404)
4. weekly	0.444 (0.786)	0.218 (0.432)
5. daily	0.407 (1.503)	0.497 (0.709)
[Q2] Alcohol-free drink status		
1. no (base level)	–	–
2. yes	0.846 * (0.485)	0.405 (0.251)
[Q4] Spending habit		
1. <€10 (base level)	–	–
2. €10–€20	0.747 (0.532)	0.464 (0.286)
3. €20–€30	0.362 (0.726)	0.298 (0.386)
4. >€30	1.187 (1.098)	0.547 (0.631)
[Q7] Preferred alcohol level in wine		
1. no preference (base level)	–	–
2. <5%	0.970 (1.130)	0.466 (0.629)
3. 5–10%	1.319 ** (0.616)	0.591 * (0.317)
4. >10%	−1.272 ** (0.580)	−0.702 ** (0.312)
[Q8] NoLo wine familiarity		
1. not familiar (base level)	–	–
2. not so familiar	1.363 ** (0.674)	0.651 * (0.333)
3. somewhat familiar	2.106 *** (0.733)	1.130 *** (0.371)
4. very familiar	4.977 *** (1.231)	2.655 *** (0.659)
5. extremely familiar	3.955 *** (1.534)	2.109 *** (0.813)
[Q9] NoLo wine tasting experience		
1. no (base level)	–	–
2. yes	3.469 *** (0.576)	1.891 *** (0.298)
[Q14] NoLo wine taste importance		
1. not important (base level)	–	–
2. not so important	−0.484 (1.427)	−0.290 (0.791)
3. somewhat important	−0.024 (1.219)	0.030 (0.658)
4. very important	0.341 (1.152)	0.257 (0.626)
5. extremely important	−0.595 (1.217)	−0.237 (0.657)
Age		
1. ≤24 (base level)	–	–
2. 25–34	−1.224 * (0.664)	−0.615 * (0.355)
3. 35–44	−2.177 * (1.125)	−1.192 ** (0.607)
4. 45–54	−0.364 (1.081)	−0.180 (0.606)
5. ≥55	−2.228 (1.841)	−1.200 (1.034)
Gender		
1. male (base level)	–	–
2. female	0.032 (0.511)	–0.016 (0.272)
3. other	−1.669 (1.362)	−0.977 (0.795)
Income		
1. no-income (base level)	–	–
2. <€15,000	0.022 (0.592)	−0.079 (0.315)
3. €15,000–€29,000	1.141 (0.767)	0.544 (0.418)
4. €30,000–€49,000	1.569 (1.018)	0.742 (0.553)
5. €50,000–€74,000	−1.805 (1.231)	−1.152 (0.691)
6. ≥€75,000	2.169 (2.144)	0.912 (1.197)
Intercept	−5.334 *** (1.401)	−2.829 *** (9.725)
N. Observations	344	344
LR $\chi^{2}$ (31)	185.63 ***	183.69 ***
Pseudo *R*^2^	0.534	0.529

Notes: For all the variables, one category was set as a base level. Coefficients for other categories are estimated relative to this base level, which is indicated by “–”. * for *p* < 0.1, ** for *p* < 0.05, *** for *p* < 0.01. Categorize with *n* < 5 have been grouped to the most adjacent one. Source: Authors’ calculations.

## Data Availability

Due to ethical considerations, GDPR protecting respondents’ personal information, and the requirements set by the Ethical Committee of the Free University of Bozen-Bolzano during the project approval process, the data obtained in this research cannot be shared publicly and may only be accessed by the authorized researchers involved in the study. Anonymized data may be obtained from the corresponding author upon reasonable request and subject to ethical approval.

## Supplementary Materials

The following supporting information can be downloaded at: https://www.mdpi.com/article/10.3390/foods15010042/s1. Table S1: Descriptive statistics and coding scheme for the main variables used in hypothesis testing and model estimation. Table S2: Association between alcohol-free drink consumption and trial experience of NoLo wines (Chi-square test). Table S3: Crosstabulation of social acceptance and willingness to recommend NoLo wines by alcohol-free beverage consumption. Table S4: Percentage of respondents who have tried dealcoholized wine by age group. The questionnaire: Exploring consumer behavior, perception, and opinion towards dealcoholized and Partially dealcoholized wine and exploring the barriers and triggers towards these wine adoption.

## References

[1] Nicholls E. (2023). “I don’t want to introduce it into new places in my life”: The marketing and consumption of no and low alcohol drinks. Int. J. Drug Policy.

[2] IWSR (2024). Consumers Show Preference for Downtrading in Beverage Alcohol, but Sentiment Is Improving in Some Markets. https://www.theiwsr.com/insight/consumers-show-preference-for-downtrading-in-beverage-alcohol-but-sentiment-is-improving-in-some-markets/.

[3] European Parliament, CEU (2021). Regulation (EU) 2021/2117 of the European Parliament and of the Council of 2 December 2021 amending Regulations (EU) No 1308/2013 establishing a common organisation of the markets in agricultural products, (EU) No 1151/2012 on quality schemes for agricultural products and foodstuffs, (EU) No 251/2014 on the definition, description, presentation, labelling and the protection of geographical indications of aromatised wine products and (EU) No 228/2013 laying down specific measures for agriculture in the outermost regions of the Union. Off. J. Eur. Union.

[4] Akhtar W., Ceci A.T., Longo E., Marconi M.A., Lonardi F., Boselli E. (2025). Dealcoholized wine: Techniques, sensory impacts, stability, and perspectives of a growing industry. Compr. Rev. Food Sci. Food Saf..

[5] Chiarini M., Calabrese F.M., Canfora I., De Boni A., Tricarico G., De Angelis M. (2025). Exploring the Multifaced Potential of Dealcoholized Wine: A Focus on Techniques, Legislation Compliances, and Microbiological Aspects. Food Rev. Int..

[6] Liguori L., Albanese D., Crescitelli A., Di Matteo M., Russo P. (2019). Impact of dealcoholization on quality properties in white wine at various alcohol content levels. J. Food Sci. Technol..

[7] An J., Zhang Z., Jin A., Tan M., Jiang S., Li Y. (2025). A Critical Review of Alcohol Reduction Methods for Red Wines from the Perspective of Phenolic Compositions. Food Sci. Nutr..

[8] Kumar Y., Ricci A., Parpinello G.P., Versari A. (2024). Dealcoholized wine: A scoping review of volatile and non-volatile profiles, consumer perception, and health benefits. Food Bioprocess Technol..

[9] Sam F.E., Ma T.-Z., Salifu R., Wang J., Jiang Y.-M., Zhang B., Han S.-Y. (2021). Techniques for dealcoholization of wines: Their impact on wine phenolic composition, volatile composition, and sensory characteristics. Foods.

[10] Gil M., Estévez S., Kontoudakis N., Fort F., Canals J., Zamora F. (2013). Influence of partial dealcoholization by reverse osmosis on red wine composition and sensory characteristics. Eur. Food Res. Technol..

[11] Corona O., Liguori L., Albanese D., Di Matteo M., Cinquanta L., Russo P. (2019). Quality and volatile compounds in red wine at different degrees of dealcoholization by membrane process. Eur. Food Res. Technol..

[12] Dumbili E.W., Leonard P., Larkin J., Houghton F. (2025). Prioritising research on marketing and consumption of No and Low (NoLo) alcoholic beverages in Ireland. Int. J. Drug Policy.

[13] Saliba A.J., Ovington L.A., Moran C.C. (2013). Consumer demand for low-alcohol wine in an Australian sample. Int. J. Wine Res..

[14] Day I., Deroover K., Kavanagh M., Beckett E., Akanbi T., Pirinen M., Bucher T. (2024). Australian consumer perception of non-alcoholic beer, white wine, red wine, and spirits. Int. J. Gastron. Food Sci..

[15] Rui M., Rosa F., Viberti A., Brun F., Blanc S., Massaglia S. Anticipating consumer preference for low-alcohol wine: A machine learning analysis based on consumption habits and socio-demographics. Proceedings of the 45th OIV Congress.

[16] Ford H., Dolan R., Goodman S., Bastian S., Pearson W., Corsi A.M. (2025). Exploring consumers’ drinking behaviour regarding no-, low-and mid-alcohol wines: A systematic scoping review and guiding framework. J. Mark. Manag..

[17] Fuentes-Fernández R., del Campo-Villares J.L. (2025). Exploring the market for dealcoholized wine in Spain: Health trends, demographics, and the role of emerging consumer preferences. Beverages.

[18] Essedielle The New Era of NoLo Wines in Italy: Understanding its Customers. https://www.essedielleenologia.com/en/news/nolo-wines-law-italy/.

[19] Stasi A., Bimbo F., Viscecchia R., Seccia A. (2014). Italian consumers׳ preferences regarding dealcoholized wine, information and price. Wine Econ. Policy.

[20] (2025). ICQRF—Cantina Italia: Report n. 8/2025; Dati al 31 Luglio 2025 dei Vini, Mosti, Denominazioni Detenuti in Italia. https://www.masaf.gov.it/flex/cm/pages/ServeBLOB.php/L/IT/IDPagina/7817.

[21] Shaw C.L., Dolan R., Corsi A.M., Goodman S., Pearson W. (2023). Exploring the barriers and triggers towards the adoption of low- and no-alcohol (NOLO) wines. Food Qual. Prefer..

[22] Kleijnen M., Lee N., Wetzels M. (2009). An exploration of consumer resistance to innovation and its antecedents. J. Econ. Psychol..

[23] Zaichkowsky J.L. (1985). Measuring the involvement construct. J. Consum. Res..

[24] Laurent G., Kapferer J.-N. (1985). Measuring Consumer Involvement Profiles. J. Mark. Res..

[25] IWSR (2024). Millennials Drive No-Alcohol Gains in the US. https://www.theiwsr.com/insight/millennials-drive-no-alcohol-gains-in-the-us/.

[26] Rogers E. (2003). Diffusion of Innovations 5th.

[27] Oliver R.L. (1980). A cognitive model of the antecedents and consequences of satisfaction decisions. J. Mark. Res..

[28] Pliner P., Hobden K. (1992). Development of a scale to measure the trait of food neophobia in humans. Appetite.

[29] Ajzen I. (1991). The theory of planned behavior. Organ. Behav. Hum. Decis. Process..

[30] Corsi A., Mazzarino S., Pomarici E. (2019). The Italian wine industry. The Palgrave Handbook of Wine Industry Economics.

[31] Bowdring M.A., McCarthy D.M., Fairbairn C.E., Prochaska J.J. (2024). Non-alcoholic beverage consumption among US adults who consume alcohol. Addiction.

[32] Rai K., Galati A., Chatterjee S., Chaudhuri R., Vrontis D., Migliore G. (2025). Uncorking the psychological factors behind habits and NoLo wine preferences. Br. Food J..

[33] Clarke N., Blackwell A.K.M., Ferrar J., De-Loyde K., Pilling M.A., Munafò M.R., Marteau T.M., Hollands G.J. (2023). Impact on alcohol selection and online purchasing of changing the proportion of available non-alcoholic versus alcoholic drinks: A randomised controlled trial. PLoS Med..

[34] Baiano A. (2025). The Willingness to Pay for Non-Alcoholic Beer: A Survey on the Sociodemographic Factors and Consumption Behavior of Italian Consumers. Foods.

[35] Masson J., Aurier P., d’Hauteville F. (2008). Effects of non-sensory cues on perceived quality: The case of low-alcohol wine. Int. J. Wine Bus. Res..

[36] Bruwer J., Jiranek V., Halstead L., Saliba A. (2014). Lower alcohol wines in the UK market: Some baseline consumer behaviour metrics. Br. Food J..

[37] Bucher T., Deroover K., Stockley C. (2018). Low-Alcohol Wine: A Narrative Review on Consumer Perception and Behaviour. Beverages.

[38] Chrysochou P. (2014). Drink to get drunk or stay healthy? Exploring consumers’ perceptions, motives and preferences for light beer. Food Qual. Prefer..

[39] Mooi E., Sarstedt M., Mooi-Reci I. (2018). Market Research.

[40] StataCorp L. (2021). Stata Meta-Analysis Reference Manual.

[41] Smith D.E., Mitry D.J. (2007). Cultural Convergence: Consumer Behavioral Changes in the European Wine Market. J. Wine Res..

[42] Ohana-Levi N., Netzer Y. (2023). Long-Term Trends of Global Wine Market. Agriculture.

[43] IWSR (2023). No-Alcohol Share of Overall Alcohol Market Expected to Grow to Nearly 4% by 2027. https://www.theiwsr.com/insight/no-alcohol-share-of-overall-alcohol-market-expected-to-grow-to-nearly-4-by-2027/.

[44] Del Rey R., Loose S. (2023). State of the International Wine Market in 2022: New market trends for wines require new strategies. Wine Econ. Policy.

[45] Cei L., Rossetto L. (2024). The demand for sparkling wine: Insights on a diversified European market. Int. J. Wine Bus. Res..

[46] Stanco M., Lerro M., Marotta G. (2020). Consumers’ Preferences for Wine Attributes: A Best-Worst Scaling Analysis. Sustainability.

[47] Palmieri N., Perito M.A. (2020). Consumers’ Willingness to Consume Sustainable and Local Wine in Italy. Y. Ital. J. Food Sci..

[48] Gow J., Moscovici D., Rana R., Rinaldi A., Ugaglia A.A., Valenzuela L., Mihailescu R., Haque R. (2024). Determinants of Purchasing Sustainably Produced Wines by Italian Wine Consumers. Sustainability.

[49] Costanigro M., Appleby C., Menke S.D. (2014). The wine headache: Consumer perceptions of sulfites and willingness to pay for non-sulfited wines. Food Qual. Prefer..

[50] Corduas M., Cinquanta L., Ievoli C. (2013). The importance of wine attributes for purchase decisions: A study of Italian consumers’ perception. Food Qual. Prefer..

[51] Johnson C.D., Kuang Y., Jankuhn N. (2020). You’re Not A Teetotaler, are You? A Framework of Nonalcoholic Wine Consumption Motives and Outcomes. J. Food Prod. Mark..

[52] Waehning N., Wells V.K. (2024). Product, individual and environmental factors impacting the consumption of no and low alcoholic drinks: A systematic review and future research agenda. Food Qual. Prefer..

[53] Bucher T., Frey E., Wilczynska M., Deroover K., Dohle S. (2020). Consumer perception and behaviour related to low-alcohol wine: Do people overcompensate?. Public Health Nutr..

[54] Lockshin L., Corsi A.M. (2012). Consumer behaviour for wine 2.0: A review since 2003 and future directions. Wine Econ. Policy.

[55] (2024). Blog. Italians’ Incomes are Growing, but Inflation is Burning Them. Portofino is the New “Capital” of Wealth, in the Cities Wide Scissors. https://excelleraint.com/i-redditi-degli-italiani-crescono-ma-l-inflazione-li-brucia-portofino-nuova-capitale-della-ricchezza/.

[56] Mosikyan S., Dolan R., Corsi A.M., Bastian S. (2024). A systematic literature review and future research agenda to study consumer acceptance of novel foods and beverages. Appetite.

[57] Günden C., Atakan P., Yercan M., Mattas K., Knez M. (2024). Consumer Response to Novel Foods: A Review of Behavioral Barriers and Drivers. Foods.

[58] Pickering G.J., Kemp B. (2024). Understanding Sparkling Wine Consumers and Purchase Cues: A Wine Involvement Perspective. Beverages.

[59] Meillon S., Urbano C., Guillot G., Schlich P. (2010). Acceptability of partially dealcoholized wines—Measuring the impact of sensory and information cues on overall liking in real-life settings. Food Qual. Prefer..

[60] Lamorte S.A., Agnoli L. Analysing consumers’ decision-making process for non-alcoholic spirit drinks and dealcoholized aromatized wines. Proceedings of the IVES Conference Series.

[61] Anderson K. (2023). The emergence of lower-alcohol beverages: The case of beer. J. Wine Econ..

[62] Perman-Howe P.R., Holmes J., Brown J., Kersbergen I. (2024). Characteristics of consumers of alcohol-free and low-alcohol drinks in Great Britain: A cross-sectional study. Drug Alcohol Rev..

[63] Times T. (2025). Italy Lifts Ban on Naming of Alcohol-Free Wines. https://www.thetimes.com/world/europe/article/non-alcoholic-wine-sold-italy-minister-g0tn27jbm.

[64] Cravero M.C., Laureati M., Spinelli S., Bonello F., Monteleone E., Proserpio C., Lottero M.R., Pagliarini E., Dinnella C. (2020). Profiling Individual Differences in Alcoholic Beverage Preference and Consumption: New Insights from a Large-Scale Study. Foods.

[65] Moffatt P. (2020). Experimetrics: Econometrics for Experimental Economics.

