# Supplementary material for: No- and Low-Alcohol Wines: Perception and Acceptance in a Traditional Wine Region in Northern Italy
Source: Foods. 2025 Dec 23;15(1):42. doi: 10.3390/foods15010042 (PMC12785501; doi:10.3390/foods15010042)
Supplement: Supplementary file 1 [file foods-15-00042-s001.zip › foods-4007835-questionnaire.pdf]

Exploring consumer behavior, perception, and opinion towards dealcoholized and Partially dealcoholized wine and exploring the barriers and triggers towards these wine adoption

The sole purpose of the questionnaires is to investigate consumer preference for dealcoholized wine as part of our research project “Study of acceptability of novel grapevine products that have lower actual alcoholic strength (dealcoholized or partially dealcoholized wines) according to the recent reg. eu 2021/2117”. Please complete the questionnaires by clicking on the checkmark box. You can choose multiple answers where required. Your answers will be kept confidential and used anonymously for research purposes in accordance with GDPR 2016/679 and the relevant national legislation. The detailed privacy notice is available on the following link:

Part I: Consumer behaviour

Q1: How often do you consume wine?

1 Daily

2 Weekly

3 Monthly

4 Occasionally

5 Rarely

Q2: Do you typically drink alcohol-free drinks?

1 Yes

2 No

Q3: Which types of wine do you prefer? (Check all that apply)

1 Red wine

2 White wine

3 Rose wine

4 Sparkling wine

5 Dessert wine

6 I don't have a preference

7 Other (please specify: ________)

Q4: On average, how much do you spend on a bottle of wine?

1 Less than €10

2 €10-€20

3 €20-€30

4 €30-€40

5 more than €40

Q5: What factors influence your decision to purchase dealcoholized wine? (Check all that apply)

1 Price

2 Brand reputation

3 Alcohol content

4 Packaging/appearance

5 Recommendations from friends/family

6 Health claims (e.g., allergies, intolerancies)

7 Vegan, organic, biodynamics, sustainability

8 Local wines/territorial wines

9 Other (please specify: ________________)

Q6: Which sensory attributes are most important to you when purchasing or evaluating wine? (Check all that apply)

1 Taste/Mouthfeel

2 Aroma

3 Appearance

4 Aftertaste

5 Other (please specify: ________________)

Q7 What is your preferred alcohol content level in dealcoholized wine?

1 Less than 5%

2 5-10%

3 10-15%

4 Over 15%

5 I have no preference

Part II: Please read the product concept and answer the following questions dealcoholized wine products

Dealcoholized or partially dealcoholized wines are varieties of wine where some or all of the alcohol content has been removed, usually through methods like distillation or membrane techniques. According to recent EU regulation, the term Dealcoholized Wine should be used for the wine products whose actual alcohol strength is not more than 0.5%, while Partially Dealcoholized Wines are wine products whose alcohol strength is above 0.5% and below the actual alcoholic strength of the product before dealcoholization.

Dealcoholized or partially dealcoholized wines are made specifically for people who want to enjoy wine flavor and aroma without or with less alcohol intake, whether for health, religious, or personal preferences. Compared to regular wine, dealcoholized or partially dealcoholized wine comes with its familiar taste, aroma, and mouthfeel, while offering no or reduced effects of alcohol.

Q8: Are you familiar of dealcoholized or partially dealcoholized wine products?

1 Extremely familiar

2 Very familiar

3 somewhat familiar

4 Not so familiar

5 Not at all familiar

Q9: Have you ever tried dealcoholized or partially dealcoholized wine?

1 Yes

2 No

Q10: If yes, Please rate your overall satisfaction with dealcoholized or partially dealcoholized wine on a scale of 1 to 10.

1 2 3 4 5 6 7 8 9 10

Q11: What are your perceptions of dealcoholized or partially dealcoholized wine? (Check all that apply)

1 Lower alcohol content

2 Healthier option

3 Less flavorful

4 Similar taste to regular wine

5 Dealcoholized or partially dealcoholized wine can be an alternative to regular wines in some occasions

6 Other (please specify: _________________)

Q12: In what situations would you be most likely to drink dealcoholized wine? (Check all that apply)

1 Casual gatherings

2 Formal dinners

3 Dealcoholized wine at non-alcoholic events

4 Relaxing at home

5 Other (please specify: _________________)

Q13: How often do you purchase dealcoholized or partially delacoholized wine?

1 Always

2 Usually

3 sometimes

4 Rarely

5 Never

Q14: How important is it to you that dealcoholized or partially dealcoholized wine tastes similar to traditional wine?

1 Extremely important

2 Very important

3 somewhat important

4 Not so important

5 Not at all important

Q15: How important is the alcohol content level when choosing dealcoholized or partially dealcoholized wine?

1 Extremely important

2 Very important

3 somewhat important

4 Not so important

5 Not at all important

Q16: How do you perceive dealcoholized or partially dealcoholized wine in terms of social acceptance?

1 Totally acceptable

2 Mostly acceptable

3 Neutral

4 Somewhat acceptable

5 Not at all acceptable

Q17: What do you expect from a high-quality dealcoholized wine? (Check all that apply)

1 Complex flavor profile

2 Smooth mouthfeel

3 Aroma complexity

4 Well-balanced acidity

5 Affordable price

6 Other (please specify: _________)

Q18: How important is it for you to have information about the production process of dealcoholized or partially dealcoholized wine?

1 Extremely important

2 Very important

3 somewhat important

4 Not so important

5 Not at all important

Q19: What would encourage you to try dealcoholized wine if you haven't already? (Check all that apply)

1 Positive reviews

2 Wine tasting opportunities

3 Discounts/promotions

4 Packaging redesign

5 Other (please specify: ________)

Q20: Which type of dealcoholized or partially dealcoholized wine would you be willing to try? (Check all that apply)

1 Dealcoholized or partially dealcoholized red wine

2 Dealcoholized or partially dealcoholized white wine

3 Dealcoholized or partially dealcoholized Rose wine

4 Dealcoholized or partially dealcoholized sparkling wine

5 Dealcoholized or partially dealcoholized dessert wine

Q21: Would you recommend dealcoholized or partially dealcoholized wine to others?

1 Definitely would

2 Probably would

3 probably would not

4 definitely would not

Demographic data

Q22: Age?

1 18-24

2 25-34

3 35-44

4 45-54

5 55-64

6 65+

Q23: Gender?

1 Male

2 Female

3 Non-binary/Other

4 Prefer not to disclose

Q24: Occupation?

1 Student

2 Teacher

3 Business owner

4 Employee

5 Retired

6 Other (please specify)

Q25: Income

1 No Income

2 Under €15,000

3 Between €15,000 and €29,000

4 Between €30,000 and €49,000

5 Between €50,000 and €74,000

6 Between €75,000 and €99,000

7 Over €100,000
