# Supplementary material for: No- and Low-Alcohol Wines: Perception and Acceptance in a Traditional Wine Region in Northern Italy
Source: Foods. 2025 Dec 23;15(1):42. doi: 10.3390/foods15010042 (PMC12785501; doi:10.3390/foods15010042)
Supplement: Supplementary file 1 [file foods-15-00042-s001.zip › foods-4007835-supplementary.pdf]

Supplementary data

Table S1. Descriptive statistics and coding scheme for the main variables used in hypothesis testing and model estimation. All single-response items were measured on the scales indicated, and higher values represent stronger levels of the measured variable. Binary variables are coded as 0 = No, 1 = Yes.

Variables	Reverse numeric coding	M	SD	Min	Max
Wine drinking frequency	(1 to 5)<br>1 = rarely to 5 = Daily	2.78	1.12	1	5
Dealcoholized familiarity	(1 to 5)<br>1 = not at all familiar to 5 = extremely familiar	1.91	1.01	1	5
Tried NoLo wines/tasting experience	(0 to 1)<br>0 = no to 1 = yes	0.28	0.45	0	1
NoLo wine purchase frequency	(1 to 5)<br>1 = never to 5 = always	1.29	0.66	1	5
Taste Importance	(1 to 5)<br>1 = Not at all important to 5 = Extremely important	3.88	1.11	1	5
Importance of alcohol content preference	(1 to 5)<br>1 = not at all important to 5 = extremely important	3.20	1.30	1	5
Perceive social acceptance	(1 to 5)<br>1 = not at all acceptable to 5 = totally acceptable	3.33	1.11	1	5
Production knowledge	(1 to 5)<br>1 = not at all important to 5 = extremely important	3.38	1.05	1	5
Willingness to Recommend	(1 to 4)<br>1 = definitely would not to 5 = definitely would	2.84	0.74	1	4

Table S2. *Association between alcohol-free drink consumption and trial experience of NoLo wines (Chi-square test).*

**Dependent variable (DV)**	**N. Categories (*K*)**	**Chi-Square (χ^2)**
Tried NoLo wines	2	0.171 (1)

Notes:
1. Statistical significance assessed with respect to $\chi^2(1)$;
2.* for $p < 0.1$, ** for $p < 0.05$, *** for $p < 0.01$.
Source: Authors' calculations

Table S3 Crosstabulation of social acceptance and willingness to recommend NoLo wines by alcohol-free beverage consumption (N = 344).

**Response category**	**Non-drinkers of alcohol-free beverages (%)**	**Drinkers of alcohol-free beverages (%)**	**Total (N)**
**Perceived social acceptance**			
**1**	76.9	23.1	13
**2**	54.8	45.2	73
**3**	47.2	52.8	106
**4**	53.8	46.2	91
**5**	36.1	63.9	61
**Total**	49.7	50.3	344
**Willingness to recommend**			
**1**	73.7	26.3	19
**2**	68.7	31.3	67
**3**	46.4	53.6	207
**4**	29.4	70.6	51
**Total**	49.7	50.3	344

Note. Values are row percentages within each response category (rounded to one decimal place). Q2 coded as 0 = non-consumer, 1 = consumer of alcohol-free drinks.

Table S3 presents the distribution of responses for social acceptance and willingness to recommend NoLo wines by alcohol-free beverage consumption. As shown, alcohol-free drink consumers were overrepresented in the highest acceptance category (63.9% vs. 36.1% non-consumers) and in the strongest recommendation category (70.6% vs. 29.4%). Conversely, non-consumers predominated in the lowest acceptance (76.9%) and lowest recommendation (73.7%) categories.

Table S4. Percentage of respondents who have tried dealcoholized wine by age group.

**Age group**	**Not tried Nolo wines (%)**	**Tried Nolo wines (%)**	**Total (n)**
1 (18-24)	77.9	22.1	172
2 (25-34)	71.7	28.3	113
3 (35-44)	61.8	38.2	34
4 (45-54)	50	50	16
5 (+55)	44.4	55.6	9
Total	72.1	27.9	344
